# Construction of a recombinant rhinovirus accommodating fluorescent marker expression

**DOI:** 10.1111/irv.12602

**Published:** 2018-09-06

**Authors:** Mingyuan Han, Charu Rajput, Joanna L. Hinde, Qian Wu, Jing Lei, Tomoko Ishikawa, J. Kelley Bentley, Marc B. Hershenson

**Affiliations:** ^1^ Department of Pediatrics & Communicable Diseases University of Michigan Medical School Ann Arbor Michigan; ^2^ Department of Molecular and Integrative Physiology University of Michigan Medical School Ann Arbor Michigan

**Keywords:** fluorescent tag, iLOV, Picornavirus, reverse genetics, rhinovirus

## Abstract

**Background:**

Rhinovirus (RV) causes the common cold and asthma exacerbations. The RV genome is a 7.3 kb single‐strand positive‐sense RNA.

**Objective:**

Using minor group RV1A as a backbone, we sought to design and generate a recombinant RV1A accommodating fluorescent marker expression, thereby allowing tracking of viral infection.

**Method:**

Recombinant RV1A infectious cDNA clones harboring the coding sequence of green fluorescent protein (GFP), Renilla luciferase, or iLOV (for light, oxygen, or voltage sensing) were engineered and constructed. RV‐infected cells were determined by flow cytometry, immunohistochemistry, and immunofluorescence microscopy.

**Results:**

RV1A‐GFP showed a cytopathic effect in HeLa cells but failed to express GFP or Renilla luciferase due to deletion. The smaller fluorescent protein construct, RV1A‐iLOV, was stably expressed in infected cells. RV1A‐iLOV expression was used to examine the antiviral effect of bafilomycin in HeLa cells. Compared to parental virus, RV1A‐iLOV infection of BALB/c mice yielded a similar viral load and level of cytokine mRNA expression. However, imaging of fixed lung tissue failed to reveal a fluorescent signal, likely due to the oxidation and bleaching of iLOV‐bound flavin mononucleotide. We therefore employed an anti‐iLOV antibody for immunohistochemical and immunofluorescence imaging. The iLOV signal was identified in airway epithelial cells and CD45+ CD11b+ lung macrophages.

**Conclusions:**

These results suggest that RV1A‐iLOV is a useful molecular tool for studying RV pathogenesis. The construction strategy for RV1A‐iLOV could be applied to other RV serotypes. However, the detection of iLOV‐expressing RV in fixed tissue required the use of an anti‐iLOV antibody, limiting the value of this construct.

## 1 INTRODUCTION

Rhinovirus (RV) is the most frequent viral infectious agent of the respiratory tract in humans and is the predominant cause of the common cold.^1^ More importantly, RV has emerged as the most frequent pathogen associated with asthma exacerbations in infants, children, and adults.^2^, ^3^, ^4^

RV is placed in the Picornaviridae family, genus Enterovirus, with three species based on phylogenetic sequence criteria.^5^, ^6^ Clinical specimens collected from in the 1960s and 1970s yielded approximately 100 different species A and B strains which were subsequently serotyped.^7^, ^8^ More recently, a diverse group of previously unrecognized human viruses from species C was found to be common causes of respiratory illness.^4^ To understand RV pathogenesis, human and animal models have been developed. Human studies have employed experimental infection with RV‐A16.^9^, ^10^ Mouse studies have used RV‐A1B wild‐type mice^11^ or RV‐A16 in mice that are transgenic for human intercellular adhesion molecule‐1.^12^ These models have been particularly useful in studying RV‐induced exacerbations of allergic airways disease. To detect RV in the tissues, investigators have employed the monoclonal antibody R16‐7.^9^, ^13^, ^14^ This antibody, originally developed by Wai‐Ming Lee at the University of Wisconsin, binds to the VP2 capsid protein of the closely related RV‐A16 and RV‐A1 strains,^6^ but not to RV‐A2, RV‐B14, or RV‐A49.^15^ Because the presence of more than 100 different RV serotypes makes it infeasible to develop a cross‐reactive antibody for RV, we sought to develop a recombinant virus with a fluorescent marker that could be used for tracking of RV infection in vivo.

Similar to other picornaviruses, RV is icosahedral, nonenveloped particle which is composed of 60 copies each of four capsid proteins, VP1, VP2, VP3, and the small myristoylated VP4.^16^, ^17^ The capsid encases a positive‐sense single‐stranded RNA (ssRNA) genome of approximately 7200 nucleotides.^18^ Following virus entry and genome release into the host cell cytoplasm, the RV ssRNA is translated into a single polyprotein that undergoes proteolytic cleavage by viral proteases 2A^pro^ and 3C^pro,^ 8, 19, 20 with the exception of the autocatalytic cleavage of precursor VP0 into VP2 and VP4 in the presence of viral RNA during the assembly process.^21^

RV infectious cDNA clones have been constructed and used as a molecular tool to study RV viral protein function and mutation‐phenotype association, as well as a vaccine vector for foreign gene expression.^17^, ^22^, ^23^, ^24^, ^25^ In the current study, we engineered a recombinant RV1A (RV1A‐iLOV) with insertion of the coding sequence for iLOV (for light, oxygen, or voltage sensing), a small‐size fluorescent marker.^26^ RV1A‐iLOV is viable, and its expressed iLOV protein is trackable both in vitro and in vivo, suggesting that RV1A‐iLOV may be a useful tool in the study of RV pathogenesis. However, the detection of iLOV‐expressing RV in fixed tissue required the use of an anti‐iLOV antibody, limiting the value of this construct.

## 2 MATERIALS AND METHODS

### 2.1 Cells and reagents

H1‐Hela and THP‐1 cells were purchased from ATCC (Manassas, VA). Plasmids pEGFP‐N1 (Clontech, Mountain View, CA), pRL (Renilla luciferase; Promega, Madison, WI), and pUC18‐ilOV (GenScript, Piscataway, NJ) were used to amplify the DNA fragments of green fluorescent protein (GFP), Renilla luciferase (RL), and iLOV, respectively. (For the detailed iLOV nucleotide sequence, see Table S1.) Antibody to RV VP2/VP0 was obtained from QED Biosciences (San Diego, CA). Anti‐GFP Ab was purchased from Thermo Fisher Scientific (Waltham, MA). A synthesized peptide fragment of iLOV (CLGRNARFLQGPETD) was generated and used to generate anti‐iLOV antibody (GenScript). Bafilomycin was purchased from Merck Millipore (Burlington, MA).

### 2.2 Design and construction of recombinant RV1A‐iLOV cDNA clone

**Figure 1:**
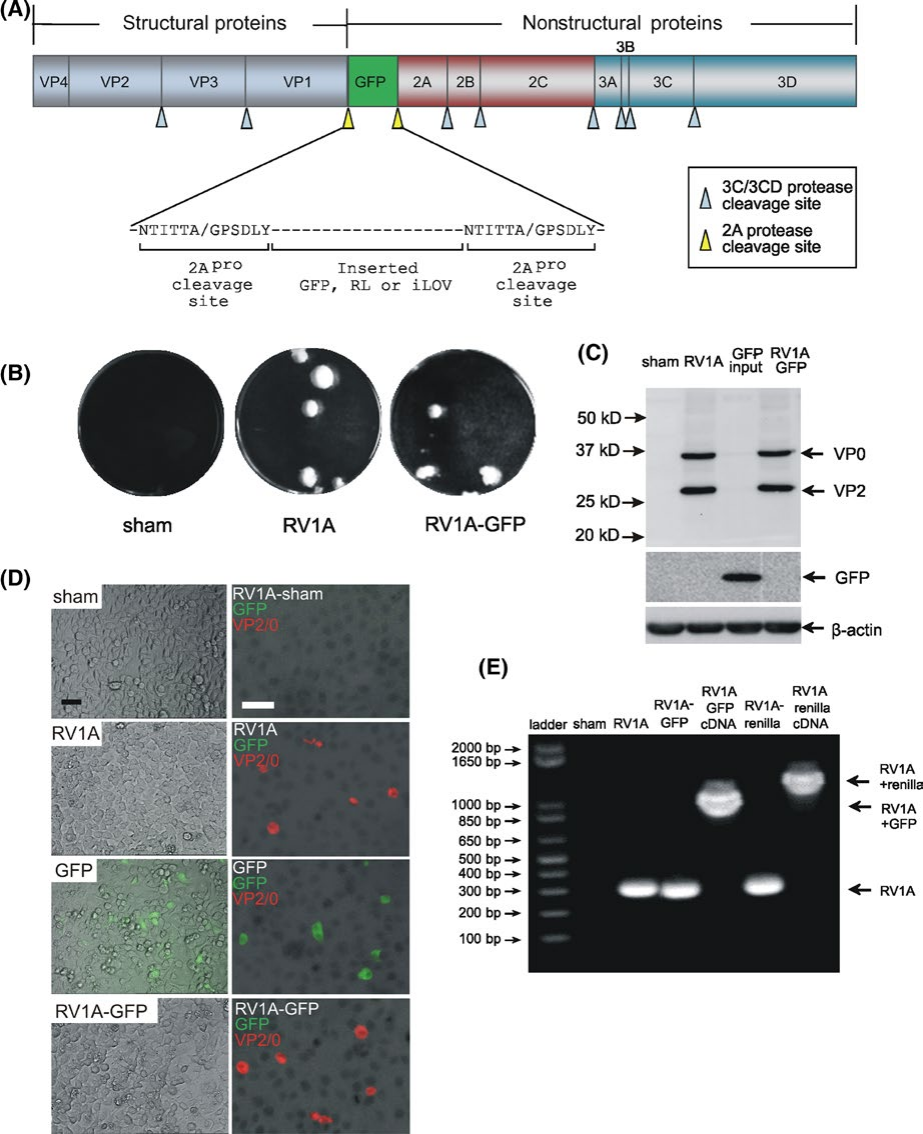
GFP ORF insertion into the rhinovirus genome is deleted. A, Schematic presentation of the insertion of GFP into RV genome. RV proteins are presented in boxes. 2A^pro^ cleavages at points indicated by yellow solid triangles separate RV structural from nonstructural proteins and releases GFP proteins. Solid blue arrows indicate 3C^pro^ cleavage sites. B, Plaque morphology of HeLa cells infected with parental wild‐type RV1A or RV1A‐GFP. C, Western blot analysis of whole‐cell lysates from HeLa cells infected with RV1A or RV1A‐GFP. Samples were probed for the presence of GFP and the RV structural proteins VP0 and VP2. GFP input was made from whole‐cell lysate of pEGFP‐N1–transfected HeLa cells. D, Detection of RV1A‐GFP–infected cells by live‐cell imaging and immunofluorescence staining. HeLa cells were infected at an MOI of 0.1 with either sham, parental‐RV1A or RV1A‐GFP for 16 h. HeLa cells were transfected with pEGFP‐N1 for 16 h (bar, 50 μm). RV VP2/0 protein was detected using AF555‐conjugated anti‐VP2/0 Ab (red); iLOV (green) was directly detected by blue laser; nuclei were stained by DAPI (shown in black; bar, 50 μm). E, RT‐PCR analysis of RV‐GFP and RV‐RL genomes. RV‐GFP and RV‐RL genomic RNA were isolated from plaque‐purified virus stocks. RV1A‐GFP and RV1A‐RL infectious cDNA clones were used as a template for amplification of complete GFP or RL sequence

The RV infectious cDNA clone encoding replication‐competent RV‐1A, pMJ3‐RV1A, was kindly provided by W. T. Jackson, University of Maryland^27^ and served as a backbone for either GFP‐, RL‐, or iLOV‐expressing viruses. GFP, RL, and iLOV open reading frames (ORFs) were designed to be flanked by the edited nucleotide sequences encoding the viral 2A^pro^ cleavage site with silent mutations introduced as described previously^24^ (Figure 1A, Table S2). Respective GFP, RL, and iLOV inserts were PCR amplified from existing clones using the primers listed in Table S1. The PCR products, which contained *Apa I* restriction enzyme cleavage sites on the 5′ and 3′ ends, were digested with *Apa I*, ligated to pMJ3‐RV1A, and transformed in *E. coli* (DH5α, Thermo Fisher Scientific). The resultant clones were sequenced to confirm the correct orientation of the inserts.

### 2.3 Generation of recombinant RV

Infectious cDNA clones encoding RV1A, RV1A‐GFP, RV1A‐RL, and RV1A‐iLOV were linearized by *Mlu I* restriction enzyme digestion. To produce replication‐competent virus, full‐length viral RNA transcripts were generated using the MEGAscript T7 Transcription Kit (Thermo Fisher Scientific) and transfected into H1‐HeLa cells using Lipofectamine MessengerMAX (Thermo Fisher Scientific). After 48 hours, cells underwent three freeze‐thaw cycles and were subjected to centrifugation at 13 800 *g* for supernatant collection. The virus‐containing supernatant stocks were designated as passage 0 (P0). H1‐HeLa cells were subsequently used to passage the virus for subsequent in vitro virus stability and in vivo studies. RV1A‐GFP and RV1A‐RL underwent plaque purification for insert analysis. RV was concentrated and partially purified from infected HeLa cell lysates by ultrafiltration using a 100 kDa cutoff filter, as described.^11^ Viral quantity was determined by plaque assay^28^ or quantitative one‐step real‐time polymerase chain reaction for positive‐strand viral RNA using RV‐specific primers and probes (forward primer: 5′‐GTGAAGAGCCSCRTGTGCT‐3′; reverse primer: 5′‐GCTSCAGGGTTAAGGTTAGCC‐3′; probe: 5′‐FAM‐TGAGTCCTCCGGCCCCTGAATG‐TAMRA‐3′).^29^ The limit of detection for this viral copy number analysis is between 0 and 10 copies. The presence of the GFP, RL, and iLOV inserts was determined using RV‐specific flanking primers (forward primer: 5′‐CATTCTGTTGTCATCACAACACA‐3′; reverse primer: 5′‐CACCTATAGTGTTTGTGCGGT‐3′). iLOV insert quantity was measured by quantitative real‐time PCR using specific primers encoding for iLOV (forward primer: 5′‐GATTCCTGCAAGGACCAGAG‐3′; reverse primer: 5′‐CCGCTCTTGGTGTAGTTGAT‐3′).

### 2.4 iLOV immunofluorescence of cultured cells

H1‐HeLa cells were infected with RV1A‐iLOV at a multiplicity of infection (MOI) of 0.1 for 24 h. Infected cells were then subjected to fluorescent microscopy. In selected experiments, RV1A‐iLOV–infected cells were fixed and stained with Alexa Fluor 555–conjugated mouse anti‐RV VP2/VP0 (clone R16‐7; QED Bioscience). Images were visualized using an Olympus IX71‐inverted phase/epifluorescence microscope and digital CCD camera.

### 2.5 Animals and RV infection

Animal usage followed guidelines set forth in the Principles of Laboratory Animal Care from the National Society for Medical Research. Six‐day‐old or 8‐ to 10‐week‐old BALB/c mice (Jackson Laboratories, Bar Harbor, ME) were treated intranasally with 15 or 50 μL of 10^8^ plaque forming units of virus and harvested 24 hours later.

### 2.6 Histology, immunohistochemistry, and immunofluorescence microscop

For histology, mouse lungs were perfused through the pulmonary artery with PBS containing 5 mmol/L EDTA and fixed with 4% paraformaldehyde overnight. For immunohistochemistry, lung sections were stained with rabbit anti‐iLOV, then incubated with biotinylated secondary goat‐IgG, ABC reagent (Vector Laboratories, Burlingame, CA), diaminobenzidine (DAB, Sigma‐Aldrich, St. Louis, MO, USA), and Gill's hematoxylin (Fisher Scientific, Kalamazoo, MI). For fluorescence microscopy, slides were incubated with Alexa Fluor 488–conjugated iLOV, Alexa Fluor 555–conjugated mouse anti‐RV VP2/VP0, and Cy5–anti‐mouse CD68 (Biolegend, San Diego, CA). Nuclei were stained with 4′,6‐diamidino‐2‐phenylindole (DAPI). Images were acquired with a Zeiss ApoTome confocal microscope (Microscopy and Image Analysis Core, University of Michigan).

### 2.7 Quantitative real‐time PCR of lung cytokines

Lung RNA was extracted with TRIzol Reagent (Thermo Fisher Scientific) combined with on‐column digestion of genomic DNA (QIAGEN, Valencia, CA). cDNA was synthesized from 1 μg of RNA and subjected to quantitative real‐time PCR using specific mRNA primers encoding for IL‐1β, IFN‐β, IFN‐γ, CXCL1, CXCL2, CXCL10, CCL2, CCL5, and IL‐10 (Table S2). For each sample, the level of gene expression was normalized to its own *GAPDH* mRNA.

### 2.8 Flow cytometric analysis

HeLa cells were infected with sham, RV1A or RV1A‐iLOV at an MOI of 0.1 for 24 hours. Cells were subjected to flow cytometry and analyzed on an LSR Fortessa (BD Biosciences, San Jose, CA). For in vivo experiments, lungs from sham‐, RV1A‐, and RV1A‐iLOV‐treated BALB/c mice were perfused with PBS containing EDTA, minced, and digested in collagenase IV. Cells were filtered and washed with RBC lysis buffer, and dead cells were stained with Pac‐Orange Live/Dead fixable dead staining dye (Fisher Scientific, Kalamazoo, MI, USA). To identify iLOV‐positive cells, cells were stained for surface markers with anti‐CD45 (BioLegend) and anti‐CD11b (Biolegend, San Diego, CA, USA). Cells were then fixed, permeabilized, and incubated with the Cy3‐tagged anti‐iLOV prior to flow cytometry. Data were collected and analyzed using FACSDiva (BD Biosciences) and FlowJo software (TreeStar, Ashland, OR).

### 2.9 Data analysis

Data are represented as mean ± SE. Statistical significance was assessed using an unpaired *t* test or one‐way ANOVA, as appropriate. Group differences were pinpointed by a Tukey's multiple‐comparison test.

## 3 RESULTS

### 3.1 Incompatibility of RV1A genome with GFP insert

GFP with flanking 2A^pro^ cleavage sites was designed to be inserted between the RV genomic sequences encoding the VP1 and 2A proteins (Figure 1A). To stabilize the genome, silent mutations were introduced into the coding sequences of both the flanking 2A^pro^ cleavage sites (Table S1), as described previously.^24^ To generate viral stocks, genomic RNA transcripts made from the RV1A‐GFP infectious clone were transfected into H1‐HeLa cells followed by three consecutive passages (P1‐P3). A cytopathic effect (CPE) was observed in HeLa cell‐infected RV1A‐GFP (Figure 1B). However, we were unable to detect GFP expression by Western blot (Figure 1C) or immunofluorescence (Figures 1D). We also constructed recombinant RV1A‐expressing Renilla luciferase (RL) protein to determine whether the size of the GFP insert exceeded the limited packaging capacity of RV. To examine the presence of intact GFP and RL ORFs from RV1A‐GFP and RV1A‐RL, RT‐PCR was performed for plaque‐purified RV1A‐GFP and RV1A‐RL using RV‐specific flanking primers. Results showed that the GFP and RL sequences were deleted (Figure 1E). Sequence analysis of inserts confirmed these results (data not shown).

### 3.2 Generation and characterization of RV1A‐iLOV

**Figure 2:**
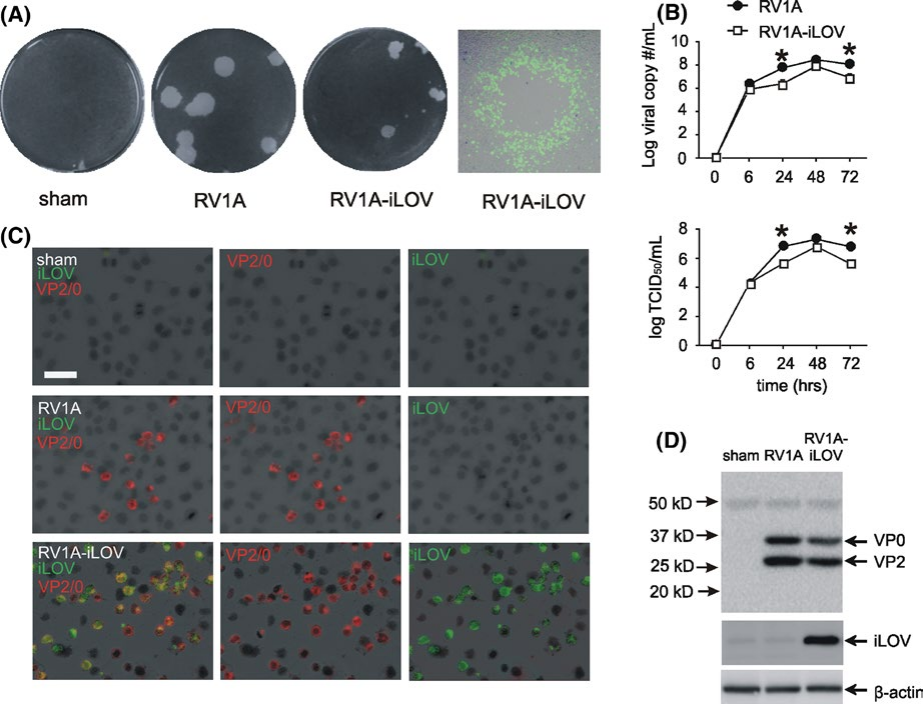
Construction of the infectious recombinant rhinovirus‐iLOV. A, Plaque morphology of HeLa cells infected with parental wild‐type RV1A or RV1A‐iLOV ***.*** iLOV (green) expression was directly detected by blue laser for RV plaques. B, Viral copy number and titers in RV‐infected HeLa cells. Cells were infected with RV1A or RV1A‐iLOV at an MOI of 0.1. At specified times, cells were harvested for analysis***.*** Viral copy number was analyzed by quantitative polymerase chain reaction. Viral titer was determined by TCID_50_ (n = 3, mean ± SD). C, Fluorescence imaging for RV‐iLOV. HeLa cells were infected with parental wild‐type RV1A or RV1A‐iLOV for 24 h at an MOI of 0.1. RV VP2/0 protein was detected using AF555‐conjugated anti‐VP2/0 Ab (red); iLOV (green) was directly detected by blue laser; nuclei were stained by DAPI (shown in black; bar, 50 μm). D, Western blot assay to detect the expression of iLOV

We reasoned that the approximately 753 nt GFP and 1005 nt RL ORFs (including engineered flanking sequences) exceeded the limited packaging capacity of RV.^24^ We therefore chose an alternative smaller fluorescent protein, iLOV (~366 nt). RV1A‐iLOV was generated following the design and generation procedure of RV1A‐GFP. CPE and growth kinetics of RV1A‐iLOV were determined in HeLa cells. In comparison with the parental virus, the RV1A‐iLOV displayed a slightly reduced cytopathic effect and growth rate (Figure 2A,B). By immunofluorescence, iLOV signal (green) appeared in RV1A‐iLOV–infected cells only, further confirming the expression of iLOV (Figure 2C). Western blot analysis using rabbit sera recognizing the iLOV protein showed expression of a product of the predicted molecular weight in RV1A‐iLOV–infected cells (Figure 2D), while viral capsid proteins VP0 and VP2 were detected in both RV1A‐ and RV1A‐iLOV–infected cells.

### 3.3 Genetic stability of RV1A‐iLOV in cell culture

**Figure 3:**
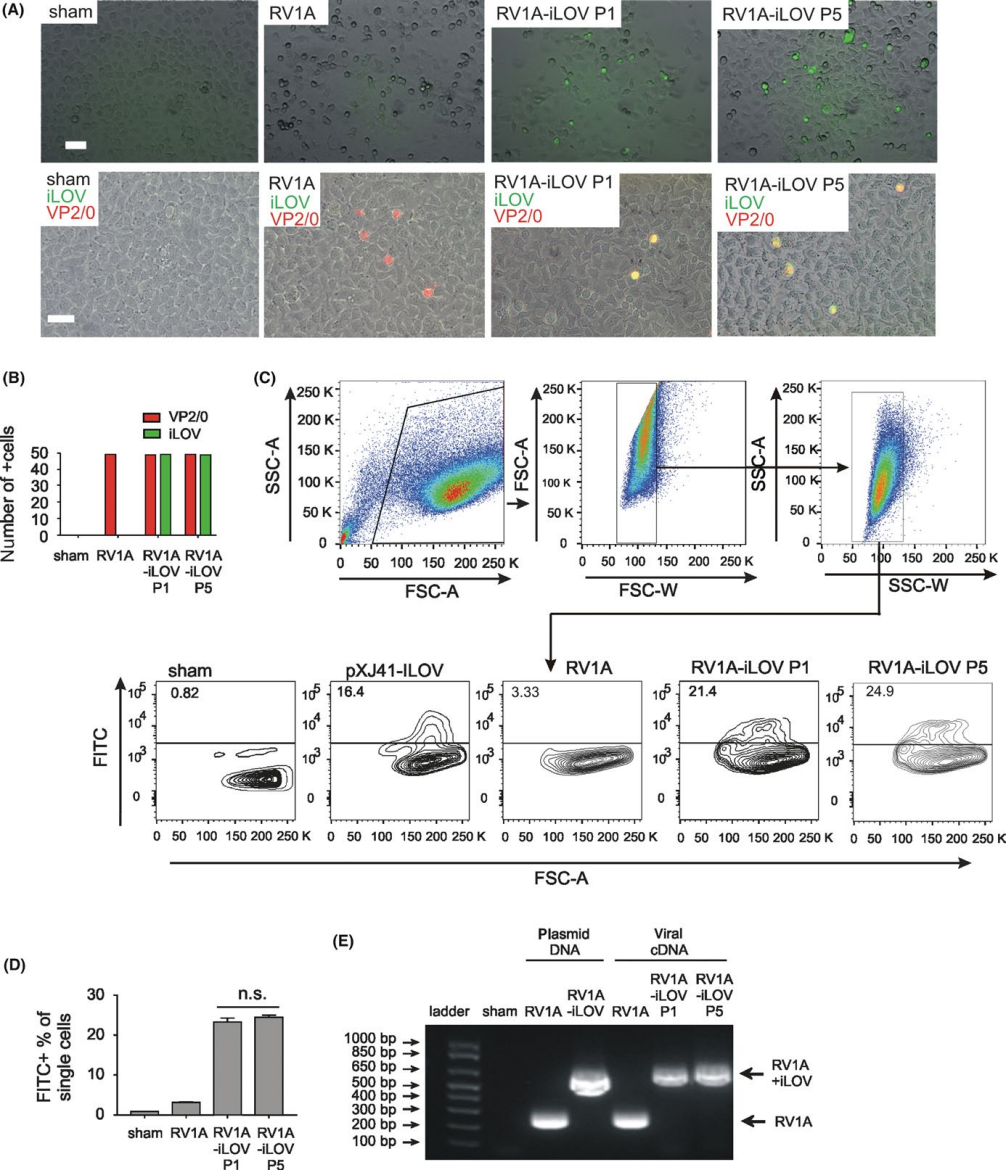
Stability of RV1A‐iLOV. A, Live‐cell imaging and immunofluorescence staining of RV1A‐iLOV–infected HeLa cells. HeLa cells were infected with P1 and P5 of RV1A‐iLOV for 24 h at an MOI of 0.1 RV VP2/0 protein, which was detected using AF555‐conjugated anti‐VP2/0 Ab (red); iLOV (green) was directly detected by blue laser; nuclei were stained by DAPI (shown in black; bar, 50 μm). B, The number of iLOV‐positive cells out of 50 VP2/0‐positive cells was counted. All VP2/0‐positive cells were iLOV‐positive. C and D, iLOV detection in HeLa cells by flow cytometry. Cells were transfected with pXJ41‐iLOV or infected with RV1A or RV1A‐iLOV, harvested 24 h later, and analyzed as a percentage of single cells (n = 3, mean ± SEM). E, RT‐PCR analysis of RV1A‐iLOV genomes. Parental RV1A or RV1A‐iLOV genomic RNA was isolated from HeLa cells infected with P1 or P5 virus stocks. The RV1A‐iLOV infectious cDNA clone was used as a template for amplification of complete iLOV sequence

Next, we examined the genetic stability of RV1A‐iLOV in HeLa cells. iLOV expression was observed in RV1A‐iLOV P1‐ and P5‐infected HeLa cells by live‐cell imaging and flow cytometry (Figure 3A‐D). All VP2/0‐positive cells were iLOV‐positive. Analysis of the P1 and P5 RV1A‐iLOV stocks by RT‐PCR revealed that exogenous iLOV DNA was stably retained within the RV genome over five passages (P1 to P5) (Figure 3E). Sequence analysis of the iLOV insert confirmed these results and revealed no mutations (data not shown).

### 3.4 Assessment of antiviral effects using RV1A‐iLOV in vitro

**Figure 4:**
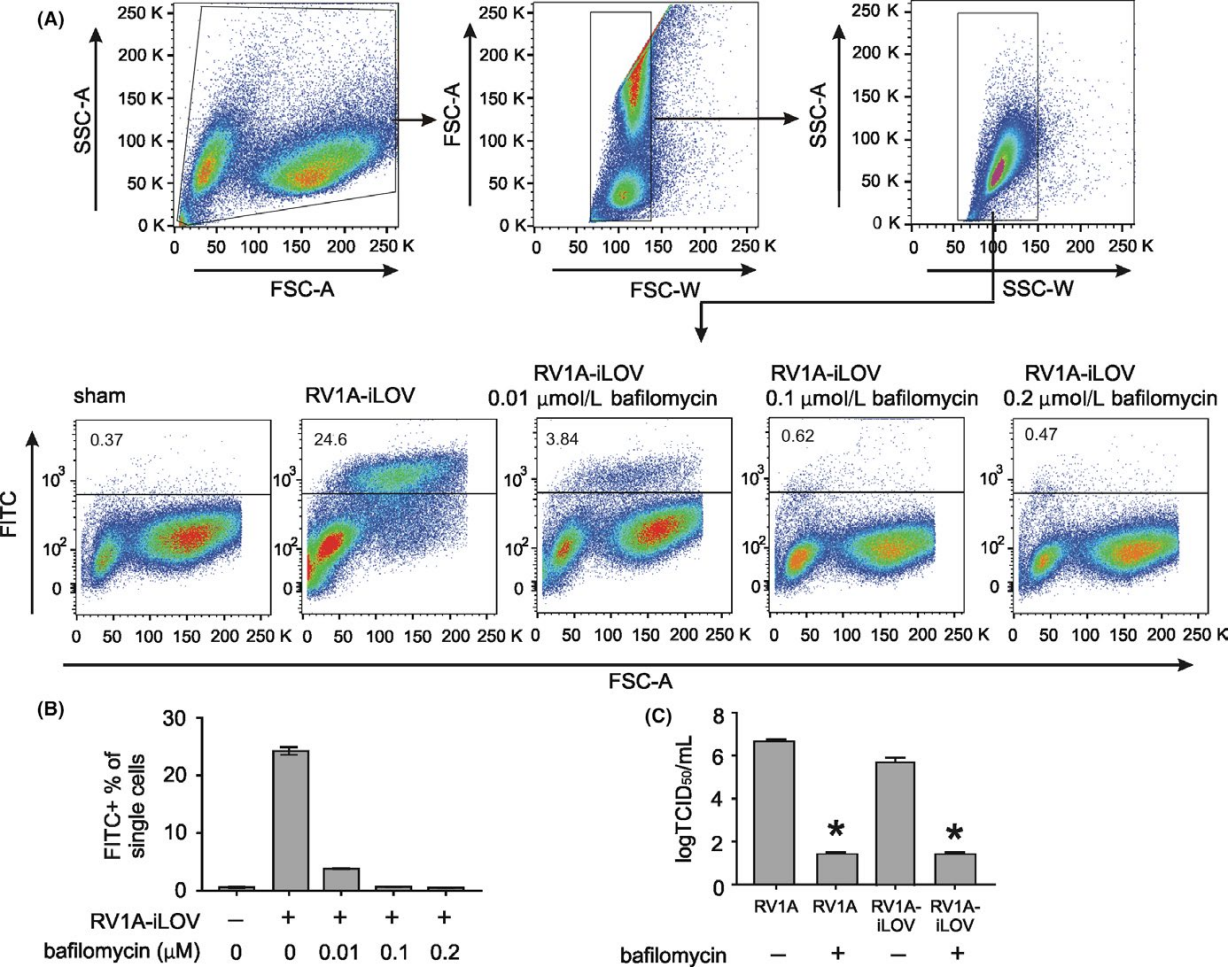
Assessment of antiviral role of bafilomycin using RV1A‐iLOV. HeLa cells were infected with sham or RV1A‐iLOV for 24 h. Selected cells were treated with 0.01 μmol/L, 0.1 μmol/L, or 0.2 μmol/L of bafilomycin. iLOV‐positive cells were analyzed as a percentage of single cells (n = 3, mean ± SEM). Viral titers were calculated as TCID_50_

We explored the application of RV1A‐ilOV to antiviral drug screening. Bafilomycin has previously been shown to inhibit RV infection.^30^ We infected HeLa cells with RV1A‐iLOV at an MOI of 0.1 for 24 hours in the presence of different concentrations of bafilomycin. iLOV expression was examined using flow cytometry (Figure 4A). Consistent with previous work,^30^ bafilomycin completely inhibited iLOV expression at a concentration of 0.1 μmol/L (Figure 4A,B) and significantly reduced the viral titers of both RV1A and RV1A‐iLOV (Figure 4C). These results demonstrate the potential utility of the iLOV construct to measure RV protein expression in vitro.

### 3.5 Induction of cytokines in RV1A‐iLOV–infected mice

**Figure 5:**
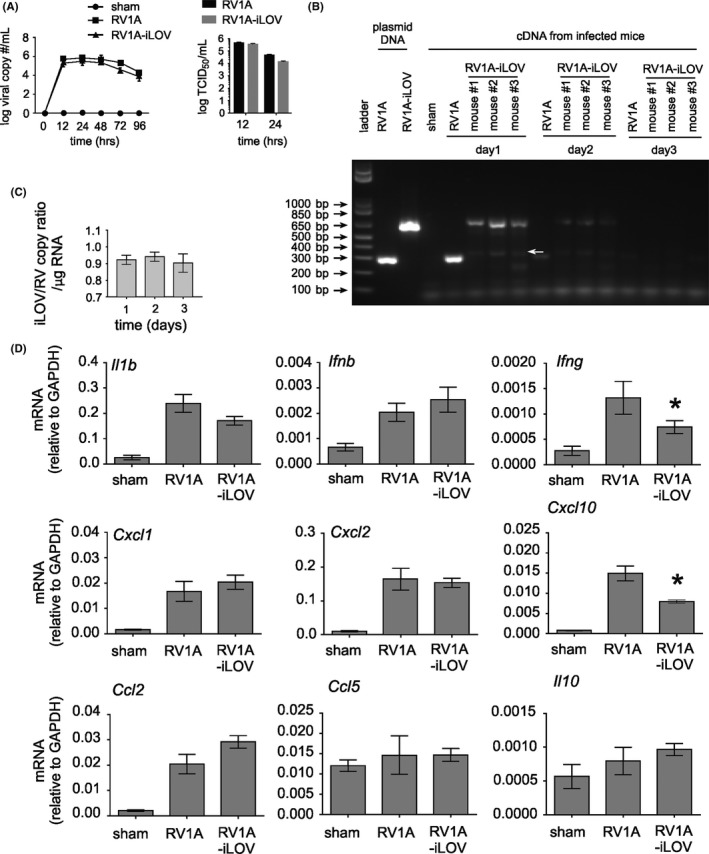
Viral load and cytokine expression of RV1A‐iLOV in vivo infection. Eight‐week‐old BALB/c mice were inoculated with sham, RV1A or RV1A‐iLOV. A, Whole lung was harvested at the indicated time points and used for measuring viral copy number and titer. B, RT‐PCR analysis of RV1A‐iLOV genomes. Parental RV1A or RV1A‐iLOV genomic RNA was isolated from infected mice at indicated time points. The RV1A‐iLOV infectious cDNA clone was used as template for amplification of complete iLOV sequence. C, iLOV/RV copy ratio in RV1A‐iLOV–infected mice. Total lung RNA (1 μg) from RV1A‐iLOV–infected mice harvested at the indicated time points was used for measuring iLOV and RV genome copy numbers. D, Whole lung mRNA expression was measured one day postinfection (N = 4, mean±SEM, *different from RV1A, one‐way ANOVA)

We previously showed RV1B, a minor group virus, triggers inflammation and cytokine expression in mice.^11^ We therefore tested whether the iLOV insert influences RV replication and RV‐induced inflammatory responses in vivo. Eight‐week‐old mice were infected with RV1A and RV1A‐iLOV for up to 4 days, and lungs were harvested at different time points after infection and processed for positive‐strand viral RNA. Measurement of RV copy number and viral titers showed no statistical difference in viral load between RV1A‐iLOV and the parental RV1A at each of the indicated time points (Figure 5A).

Studies in coxsackievirus have shown that large insertions at the analogous capsid protein P‐1D protease 2A junction may delete readily.^31^, ^32^ iLOV stability in vivo was therefore examined by RT‐PCR at each of the indicated time points (Figure 5B). The intact iLOV fragment (~600‐bp band) along with a size‐reduced band (~300 bp) appeared in RV1A‐iLOV–infected mice. Sequence analysis of the size‐reduced band revealed the deletion of the coding sequence for intact iLOV plus three nucleotides from 2A^pro^ (see sequence in Table S1). Retention of the iLOV insert was approximately 90% at the indicated time points (Figure 5B,C).

We next examined cytokine mRNA expression in RV1A‐iLOV–infected mice. Lungs were harvested one day after infection. Similar to the parental RV1A virus, RV1A‐iLOV increased lung mRNA levels of *Il1b, Ifnb1, Ifng, Cxcl1, Cxcl10, Cxcl2,* and *Ccl2* (Figure 5D). However, *Ifng* and *Cxcl10* mRNA expressions were decreased for RV1A‐iLOV compared to RV1A, perhaps because of the slightly reduced growth rate. No induction of *Ccl5* or *Il10* was observed for either RV1A or RV1A‐ilOV.

### 3.6 Detection of iLOV in lungs of infected mice

**Figure 6:**
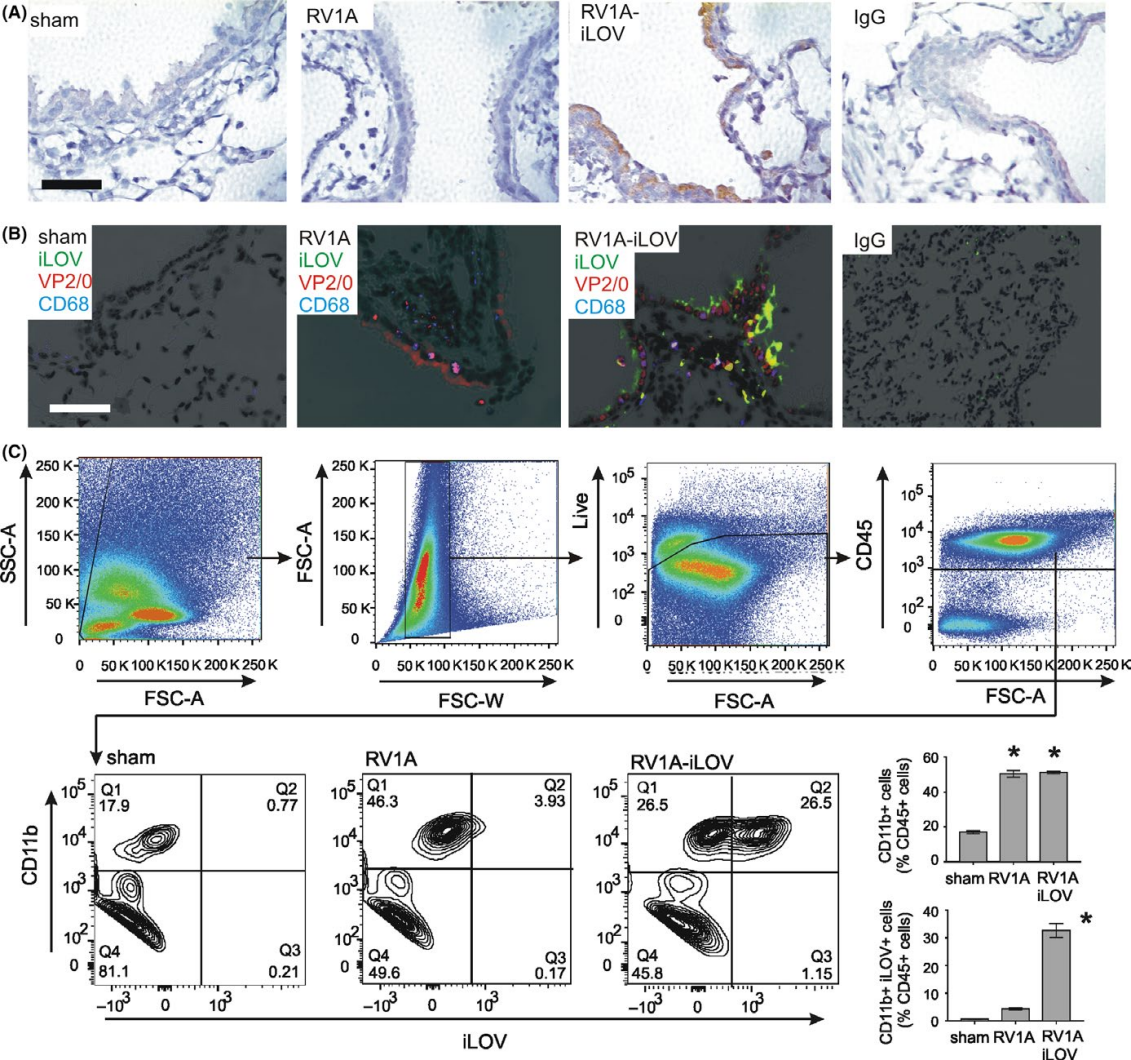
Presence of iLOV signal in the airway epithelium and lung macrophages of RV1A‐iLOV–infected mice. A,Twenty‐four hours after infection, lungs were fixed in formaldehyde overnight, embedded in paraffin, sectioned at 5 μm, and incubated with a 1:1000 dilution of anti‐iLOV or isotype control IgG (bar, 50 μm). B, Lung sections were costained with AF‐488–conjugated anti‐iLOV (green), AF‐555–conjugated anti‐VP2/0 (red), and Cy5‐conjugated CD68 (far red optical spectrum, shown in blue). C, Lung CD45 + , CD11b+, iLOV+ cells from RV‐infected BALB/c mice were identified one day postinfection and analyzed as a percentage of CD45 +  cells (n = 4 from one experiment). Data are presented as mean ± SEM (*different from sham, *P* < 0.05; one‐way ANOVA)

HeLa cells were plated on coverslips, infected with RV1A‐iLOV, and fixed in 4% paraformaldehyde. A fluorescent signal was visualized, but the signal was rapidly quenched, likely due to the oxidation and bleaching of iLOV‐bound flavin mononucleotide. Similarly, imaging of fixed lung tissue from RV1A‐iLOV–infected mice failed to reveal a fluorescent signal. We therefore employed an anti‐iLOV antibody for immunohistochemical staining and immunofluorescence imaging. We infected mice with RV1A‐iLOV and harvested lungs one day postinfection. We have previously shown that, besides airway epithelial cells, RV colocalizes with CD68+ macrophages.^13^, ^14^ Immunohistochemical staining with anti‐iLOV showed signal in both the epithelium and macrophages (Figure 6A). Immunofluorescence similarly indicated localization in the airway epithelium, as well as colocalization of iLOV (green) with VP2/0 (red) and a macrophage marker, CD68 (blue, Figure 6B).

We have previously shown that RV infection induces lung infiltration with CD11b‐positive, M2‐polarized exudative macrophages.^33^ For the analysis of intracellular iLOV, aliquots of lung mince were fixed, permeabilized, and incubated with the Cy3‐tagged anti‐iLOV prior to flow cytometry. Flow cytometric analysis showed similar increases in the percentage for CD45 +  CD11b+ cells in both RV1A‐ and RV1A‐iLOV–infected mice (Figure 6C), confirming the colocalization of lung macrophages and RV. However, a signal was detected in CD45+ CD11b+ cells only in RV1A‐iLOV–infected mice. Taken together, these results suggest RV1A‐iLOV as a potential tool to study RV‐induced responses in immature mice.

## 4 DISCUSSION

In the present study, we sought to design and generate a recombinant RV1A accommodating fluorescent marker expression, thereby allowing tracking of viral infection in vivo. Using reverse genetics, we engineered and constructed recombinant RV1A infectious cDNA clones harboring the coding sequences of GFP, RL, or iLOV. GFP and RL were not expressed in cultured cells due to deletion during replication, consistent with the limited packaging capability of other picornaviruses. On the other hand, the smaller fluorescent protein construct, iLOV, was stably expressed in RV1A‐iLOV–infected cells both in vitro and in vivo. Evaluation of iLOV expression was used to assess the antiviral effects of bafilomycin in RV1A‐iLOV–infected cells in vitro. Further, in vivo studies showed that, compared to parental virus, RV1A‐iLOV yielded a similar viral load and level of cytokine mRNA expression in the lungs of infected mice. These results suggest RV1A‐iLOV may be a useful molecular tool for studying the life cycle and pathogenesis of RV.

Construction of recombinant viruses expressing fluorescent markers, especially GFP, has been applied through reverse genetics to RNA viruses including influenza virus,^34^ Zika virus,^35^ West Nile virus,^36^ respiratory syncytial virus,^37^ murine coronavirus,^38^ and porcine reproductive and respiratory syndrome virus.^39^ However, insertion of large fluorescent protein coding sequences into the genome of the picornaviruses, a group of small RNA viruses whose genome sizes range from 7.2 to 8.5 kb, has been problematic. Insertion of the GFP ORF into poliovirus severely impaired viral replication and was deleted in the course of cell culture serial passage.^40^ In addition, an attempt to construct a recombinant foot‐and‐mouth disease virus (FMDV) expressing GFP or Renilla luciferase protein failed, likely due to the limited packaging capability.^41^ Subsequent construction of viruses containing increasingly larger inserts suggested 300–400 nt as the maximum size to be inserted into FMDV genome. Consistent with this, RV1A has been used to express a 393‐nt–long fragment of HIV gag gene.^24^ Shorter antigenic tags have also been inserted into the nonstructural proteins of poliovirus.^42^

Compared to GFP, fluorescent proteins based on flavin‐binding LOV (light, oxygen, or voltage sensing) domain offer advantages owing to their smaller size (354 nt), pH, and thermal stability.^43^ iLOV was created from the LOV2 domain of the phototropin 2 plant blue light receptor of *Arabidopsis thaliana*.^26^ Unlike GFP‐based fluorescent proteins which are inherently fluorescent, LOV domains specifically function as photosensory modules and typically bind flavin mononucleotide as an ultraviolet blue light‐absorbing chromophore. Accordingly, iLOV has been used as a reporter gene in recombinant FMDV^41^ and reovirus.^44^ However, in the latter studies, iLOV expression was only examined in cultured mammalian cells, not in vivo experiments. We now show that iLOV is expressed in RV1A‐iLOV–infected cells in vivo. Though some deletion occurs during in vivo infection, over 90% of the recombinant RV1A‐iLOV retains the iLOV sequence. However, while iLOV was readily detectable in cultured HeLa cells, the iLOV fluorescence was rapidly lost in fixed cells and lung tissue, likely due to the oxidation and bleaching of iLOV‐bound flavin mononucleotide. We therefore required the use of anti‐iLOV antibody to detect RV1A‐iLOV.

Similar to viral proteins, iLOV protein is released from the RV polyprotein through viral proteinase‐mediated auto‐cleavage during viral protein production, an early step of viral replication. Detection of iLOV in mouse tissue is therefore highly suggestive of viral replication, particularly in the airway epithelium. However, it remains unclear whether iLOV signal in macrophages represents replication or engulfment of the virus by phagocytosis. Viral replication in cultured macrophages is limited,^45^ though it has recently been shown that airway epithelial cells promote rhinovirus replication in monocytic cells.^46^

Because there are more than 100 different RV serotypes (in species A and B alone), it is infeasible to develop a cross‐reactive antibody for RV. Until now, only one antibody has been available for this purpose, the monoclonal antibody R16‐7. This antibody binds to the VP2 capsid protein of the closely related RV‐A16 and RV‐A1 strains^6^ but not to RV‐A2, RV‐B14, or RV‐A49.^15^ We developed a recombinant virus with a fluorescent marker that could be used for tracking of RV infection in vivo. We designed the iLOV sequence to be flanked with 2A^pro^ cleavage sites and then inserted between the RV genomic sequences encoding the VP1 and 2A proteins. 2A^pro^ mediates auto‐cleavage between VP1 and 2A proteins.^20^, ^47^ Since self‐catalytic cleavage is a characteristic of picornavirus replication, this design should allow extension of our technique to all human RVs. Given the fact that iLOV sequence was stably maintained within RV1A genome during consecutive passages, it is plausible that other RVs serotypes would accommodate and maintain the stability of iLOV sequence. Though the 2A^pro^ cleavage sites of numerous RV serotypes are heterogeneous,^48^ designing the flanked 2A^pro^ cleavage sequence to be serotype‐specific would guarantee the release of iLOV. Besides VP1‐2A cleavage site, the junction site between 5′UTR and the N‐terminus of VP4 has been used to insert GFP in the genome of coxsackie A16 virus^49^; however, the insertion impaired viral replication. Taken together, these data suggest that the construction strategy for RV1A‐iLOV could be applied to other RV serotypes for studying the life cycle of RV in cultured cells, screening for antiviral drugs and for the pathogenesis of RV.
