# Supplementary material for: Construction of a recombinant rhinovirus accommodating fluorescent marker expression
Source: Influenza Other Respir Viruses. 2018 Sep 6;12(6):717–27. doi: 10.1111/irv.12602 (PMC6185886; doi:10.1111/irv.12602)
Supplement: Supplementary file 1 [file IRV-12-717-s001.docx]

**Supplementary data:**

**Construction of a recombinant rhinovirus accommodating fluorescent marker expression**

Mingyuan Han^1^, Charu Rajput^1^, Joanna L. Hinde^1^, Qian Wu^1^, Jing Lei^1^, Tomoko Ishikawa^1^, J. Kelley Bentley^1^, Marc B. Hershenson^1,2^

Departments of Pediatrics & Communicable Diseases and Molecular and Integrative Physiology, University of Michigan Medical School, Ann Arbor, MI 48109

**Table S1. Recombinant iLOV sequences.**

**iLOV nucleotide sequence:** *GGGCCC***TCC**GA**C**CT**C**TA**C**ATCGAGAAGAACTTCGTGATCACCGACCCCAGACTGCCCGACAACCCCATCATCTTCGCCAGCGACGGCTTCCTGGAGCTGACCGAGTACAGCAGAGAGGAGATCCTGGGCAGAAACGCCAGATTCCTGCAAGGACCAGAGACCGACCAGGCCACCGTGCAGAAGATCAGAGACGCCATCAGAGACCAGAGAGAGACCACCGTGCAGCTGATCAACTACACCAAGAGCGGCAAGAAGTTCTGGAACCTGCTGCACCTGCAGCCCGTGAGAGACCAGAAGGGCGAGCTGCAGTACTTCATCGGCGTGCAGCTGGACGGCAGCGACCACGTGAA**T**AC**A**AT**C**AC**C**AC**A**GC**C***GGGCCC*

(Flanked 2A^pro^ cleavage site are underlined; introduced silent mutations are shown in bold font; *Apa I* restriction enzyme cleavage site is italicized.)

**Recombinant iLOV amino acid sequence:**

GPSDLYIEKNFVITDPRLPDNPIIFASDGFLELTEYSREEILGRNARFLQGPETDQATVQKIRDAIRDQRETTVQLINYTKSGKKFWNLLHLQPVRDQKGELQYFIGVQLDGSDHVNTITTAGP

(Flanked 2A^pro^ cleavage sites are underlined.)

**Deleted sequence in RV1A-iLOV infected mice:**

*GGGCCC***TCC**GA**C**CT**C**TA**C**ATCGAGAAGAACTTCGTGATCACCGACCCCAGACTGCCCGACAACCCCATCATCTTCGCCAGCGACGGCTTCCTGGAGCTGACCGAGTACAGCAGAGAGGAGATCCTGGGCAGAAACGCCAGATTCCTGCAAGGACCAGAGACCGACCAGGCCACCGTGCAGAAGATCAGAGACGCCATCAGAGACCAGAGAGAGACCACCGTGCAGCTGATCAACTACACCAAGAGCGGCAAGAAGTTCTGGAACCTGCTGCACCTGCAGCCCGTGAGAGACCAGAAGGGCGAGCTGCAGTACTTCATCGGCGTGCAGCTGGACGGCAGCGACCACGTGAA**T**AC**A**AT**C**AC**C**AC**A**GC**C***GGGCCC*

(Flanked 2A^pro^ cleavage sites are underlined; introduced silent mutations are shown in bold font; *Apa I* restriction enzyme cleavage site is italicized; the deleted sequence is in blue font.)

**Table S2. Primer sets for GFP, RL and iLOV inserts.**

Primers Sequence (5’-3’)

RV-iLOV (Forward) ATGGGGCCCTCCGACCTCTACATCGAGAAGAACTTCGTGATCACCG

RV-iLOV (Reverse) ATCGGGCCCGGCTGTGGTGATTGTATTCACGTGGTCGCTGCCGTCCAGCT

RV-GFP (Forward) GCTGGGCCCTCCGACCTCTACATGGTGAGCAAGGGCGAGGAGCTG

RV-GFP (Reverse) ACTGGGCCCGGCTGTGGTGATTGTATTCTTGTACAGCTCGTCCAT

RV-RL (Forward) GCTGGGCCCTCCGACCTCTACATGACTTCGAAAGTTTATGATCCAG

RV-RL (Reverse) ACTGGGCCCGGCTGTGGTGATTGTATTTTGTTCATTTTTGAGAACTCGCT

Underlined: flanked 2A^pro^ cleavage site

**Table S3. Primer sets for qPCR analysis**.

Primers Sequence

MouseIFN-β (Forward) 5’-GACGGAGAAGATGCAGAAGAGTTAC-3’

MouseIFN-β (Reverse) 5’-CCACCCAGTGCTGGAGAA-3’

MouseIFN-γ (Forward) 5’-TGGCTGTTTCTGGCTGTTAC-3’

MouseIFN-γ (Reverse) 5’-TCCACATCTATGCCACTTGAGTT-3’

MouseIL-1β (Forward) 5’-TGGCAGCTACCTGTGTCTTTC-3’

MouseIL-1β (Reverse) 5’-GGATGGGCTCTTCTTCAAAGATG-3’

MouseCXCL-1 (Forward) 5’- TGCACCCAAACCGAAGAAGTCAT-3’

MouseCXCL-1 (Reverse) 5’- CAAGGGAGCTTCAGGGTCAAG-3’

MouseCXCL-2 (Forward) 5’- GCGCTGTCAATGCCTGAAG-3’

MouseCXCL-2 (Reverse) 5’- CGTCACACTCAAGCTCTGGAT-3’

MouseCXCL-10(Forward) 5’- GCTGCAACTGCATCCATATC-3’

MouseCXCL-10 (Reverse) 5’- TTTCATCGTGGCAATGATCT-3’

MouseCCL-2(Forward) 5’- GCTCTCTCTTCCTCCACCAC-3’

MouseCCL-2(Reverse) 5’- GCGTTAACTGCATCTGGCT-3’

MouseCCL-5(Forward) 5’- TGGCTCGGACACCACTCCCTG-3’

MouseCCL-5(Reverse) 5’- ACTCCTTGACGTGGGCACGAG-3’

MouseIL-10(Forward) 5’- GCTCTTACTGACTGGCATGAG-3’

MouseIL-10(Reverse) 5’ CGCAGCTCTAGGAGCATGTG-3’
